# A Crunch That Spoke: An Atypical Presentation of Spontaneous Pneumomediastinum in an Elderly Male

**DOI:** 10.7759/cureus.113314

**Published:** 2026-07-24

**Authors:** Manisha Shree B, Gomathi Karmegam, Raja Subramanian, Ashwini G, Pavithra R, Shalini K, Harishri Suresh Babu, Sahasyaa Adalarasan

**Affiliations:** 1 Emergency Medicine, Madras Medical College, Chennai, IND; 2 Medicine, Madras Medical College, Chennai, IND

**Keywords:** causes of pneumomediastinum, crepitus, hamman's crunch, hamman’s syndrome, spontaneous pneumomediastinum (spm), subcutaneous emphysema management

## Abstract

Spontaneous pneumomediastinum is a rare, usually self-limiting, condition characterized by the presence of free air in the mediastinal cavity without any identifiable precipitating factor. We report the case of a 63-year-old man who presented with chest pain, chest tightness, and shortness of breath. The patient had no known comorbidities and no history of smoking or alcohol intake. On examination, the patient was found to have subcutaneous emphysema around the neck and chest progressing to the face and upper limbs and extending to the groin, with no external injuries. Crepitus was present, extending from the face to the abdomen, and Hamman’s crunch was noted. Chest imaging revealed free air within the mediastinum, confirming the diagnosis of spontaneous pneumomediastinum. The patient was treated with high-flow oxygen via a non-rebreather mask, and the respiratory condition was closely monitored. A left-sided intercostal drainage tube was placed due to extensive subcutaneous emphysema. The patient gradually showed clinical improvement and was discharged following the resolution of pneumomediastinum.

## Introduction

Pneumomediastinum is characterized by the presence of free air in the mediastinum. It is often a result of physical trauma or other situations that may cause the leakage of air from the lungs, airways, or bowel into the mediastinum [1]. Mediastinum refers to the central visceral compartment of the thoracic cavity, bounded laterally by the parietal pleura, superiorly by the thoracic inlet, inferiorly by the diaphragm, and posteriorly by the thoracic vertebral column. Pneumomediastinum can be classified into spontaneous pneumomediastinum and secondary pneumomediastinum [2]. In cases without any apparent traumatic cause or identifiable precipitating factor, it is referred to as spontaneous pneumomediastinum or Hamman’s syndrome [1].

Idiopathic spontaneous pneumomediastinum is a rare, usually self-limiting condition that commonly affects young adult males. It was first described by Louis Hamman in 1939, along with the characteristic precordial crepitus synchronous with the heartbeat, now known as Hamman’s sign [3]. Common symptoms associated with pneumomediastinum include chest pain, dyspnea, and subcutaneous emphysema [4]. Subcutaneous emphysema is a condition in which air gets trapped under the skin and in the soft tissues, and it is associated with swelling around the neck region [5].

Factors like recreational drug use and smoking may contribute to spontaneous pneumomediastinum [6,7]. The suspected pathophysiology of pneumomediastinum is commonly explained by the Macklin effect, first described by Macklin in 1944. Increased intra-alveolar pressure, commonly due to coughing, inhalation injury, forceful retching or vomiting, results in alveolar rupture, and air moves along the peribronchial and perivascular sheaths into the mediastinum [8,9].

Chest X-ray (CXR) is commonly used to make a diagnosis of pneumomediastinum, although Kaneki et al. found chest CT to be more effective than CXR alone to diagnose pneumomediastinum [10]. Common radiological signs include the continuous diaphragm sign caused by the presence of posterior pericardial air, the thymic sail sign produced by elevation of the thymus by mediastinal air, and the extra-plural air sign resulting from air tracking between the parietal pleura and diaphragm [2,9].

## Case presentation

A 63-year-old male presented to the outpatient department with acute chest symptoms. The patient had complaints of chest pain, chest tightness, and shortness of breath for one day. There was no history of smoking or alcohol consumption and no known comorbidities. The patient was conscious, oriented to time and place, and dyspneic. On examination, the patient was found to have subcutaneous emphysema around the neck and chest progressing to the face and upper limbs, and crepitus extending from the face to the abdomen. No external injuries were found, and Hamman’s sign was present. Heart rate was recorded as 100 bpm, blood pressure 130/90 mmHg, and oxygen saturation was only 85% on room air, which improved to 98% when placed on 12L O_2_ via a non-rebreather mask (NRBM). The patient had no history of trauma, vomiting, or strenuous activity, and no binge alcohol intake.

**Figure 1:**
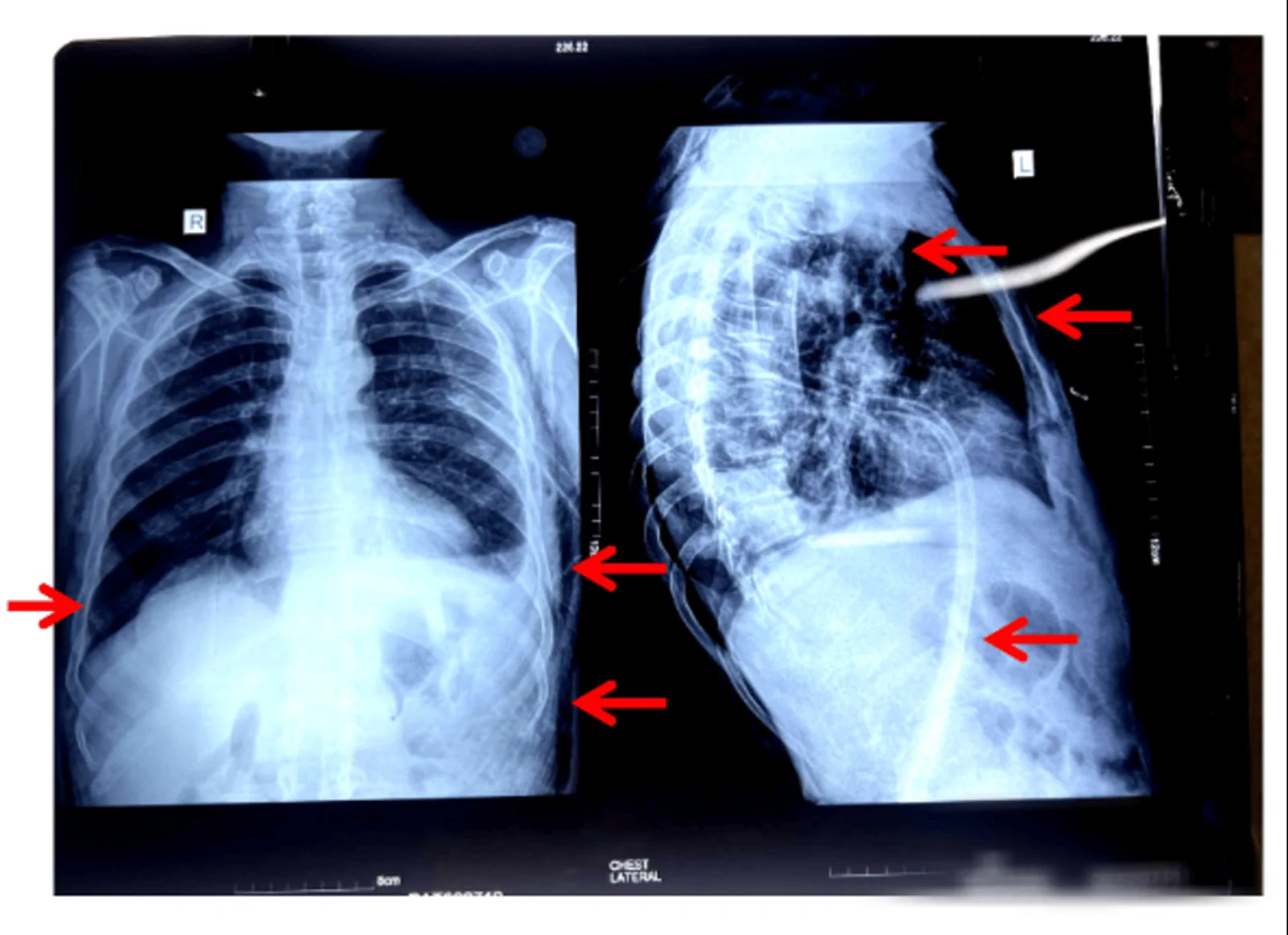
Chest X-ray showing pneumomediastinum with subcutaneous emphysema Posteroanterior chest radiograph demonstrating extensive pneumomediastinum with arrows representing subcutaneous emphysema involving the cervical and thoracic soft tissues. Additionally, no evidence of pneumothorax is identified.

**Figure 2:**
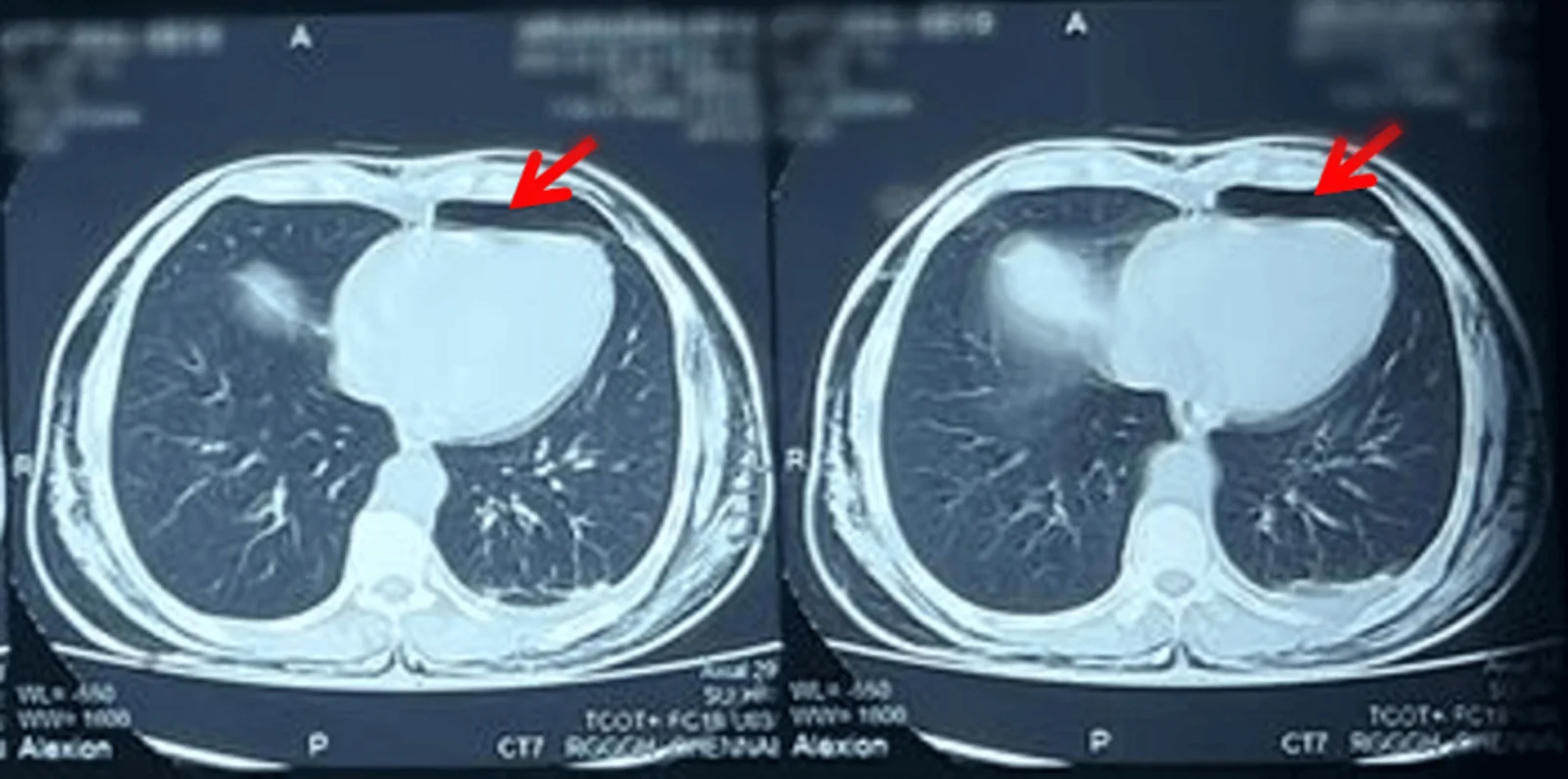
CT chest showing pneumomediastinum Axial computed tomography (CT) image of the chest, with arrows showing extensive free air within the mediastinum, confirming pneumomediastinum.

The patient was initially evaluated for several differential diagnoses, including Hamman’s syndrome, Boerhaave’s syndrome, pulmonary embolism, pneumothorax, and aortic dissection. Investigations, including electrocardiography (ECG), arterial blood gas (ABG), and complete blood count (CBC), were all found to be normal. The absence of ECG abnormalities and hemodynamic instability reduced the likelihood of pulmonary embolism. Chest X-ray demonstrated pneumomediastinum with subcutaneous emphysema, without evidence of pneumothorax (Figure 1). Boerhaave’s syndrome was considered less likely in view of the absence of vomiting, retching, or alcohol use. Aortic dissection was also considered unlikely as the patient did not have a widened pulse pressure, pulse deficits, or radiological evidence suggestive of dissection. A CT chest was performed to confirm the pneumomediastinum and subcutaneous emphysema. Free air was visualized in the mediastinum on the CT (Figure 2). A diagnosis of spontaneous pneumomediastinum (Hamman’s syndrome) with subcutaneous emphysema was confirmed.

The patient was managed with high-flow oxygen via an NRBM and was closely monitored for respiratory distress, with multidisciplinary consultation from Cardiothoracic and Vascular Surgery and Respiratory Medicine. The patient had progressive subcutaneous emphysema involving the face, bilateral upper limbs, and extending to the groin, for which a left-sided intercostal drainage tube was placed. Gradual clinical improvement was noted in the patient, and subcutaneous emphysema resolved within one week. Following the resolution of pneumomediastinum, the patient was discharged in stable condition.

## Discussion

Pneumomediastinum is the presence of free air within the mediastinal cavity. It may occur spontaneously or secondary to trauma, airway injury, or underlying lung pathology. Spontaneous pneumomediastinum (Hamman’s syndrome) is a rare, usually self-limiting condition seen predominantly in young males, commonly presenting with chest pain, dyspnea, and subcutaneous emphysema. The suspected pathophysiology in pneumomediastinum is commonly explained by the Macklin effect, in which increased intra-alveolar pressure leads to alveolar rupture and air leakage into the mediastinum.

Susai et al. highlighted the importance of computed tomography, especially when chest radiography is inconclusive or when complications are suspected [2]. In our patient, the chest radiograph demonstrated pneumomediastinum with subcutaneous emphysema, while CT confirmed the diagnosis and more precisely defined the extent of mediastinal and subcutaneous air, facilitating appropriate clinical management.

The patient presented with chest pain, chest tightness, and shortness of breath, and was found to have subcutaneous emphysema around the neck and chest progressing to the face and upper limbs. Crepitus was present from the face to the abdomen. Morgan et al. reported that most patients with primary spontaneous pneumomediastinum have identifiable precipitating factors, such as smoking, cough, asthma, physical exertion, and retching, none of which were present in our patient [11].

A systematic review of 339 cases of spontaneous pneumomediastinum conducted by Alemu et al. noted that the mean age of patients affected with spontaneous pneumomediastinum was 22.4 ± 11.3 years, highlighting the unusual presentation in our elderly patient, who was 63 years old. Chest pain was found to be the most common presenting symptom, seen in 57.8% of cases, along with dyspnea (46%), cough (28%), and neck swelling (27.13%). Similar to the findings in the review, our patient presented with acute chest pain, dyspnea, and extensive subcutaneous emphysema. The mean duration for clinical resolution of spontaneous pneumomediastinum was reported to be 6.65 ± 11.8 days, comparable to our case, which was resolved within a week. Hamman's sign is regarded as a classic but relatively uncommon physical finding in spontaneous pneumomediastinum, reported in only 11.2% of the total cases; however, it was clearly seen in our patient, illustrating the continued clinical value of careful physical examination despite widespread availability of advanced imaging [12].

Generally, spontaneous pneumomediastinum with subcutaneous emphysema follows a self-limiting course, and no specific therapy is needed [13]. Consistent with the recommendations of Takada et al., our patient was managed conservatively with oxygen therapy and close observation [14]. However, the extensive progression of subcutaneous emphysema necessitated intercostal drainage, illustrating that individualized management may occasionally be required.

The patient was treated with high-flow oxygen through an NRBM and was kept under close observation for signs of respiratory distress. Following treatment, the patient showed gradual clinical improvement and was discharged in stable condition.

## Conclusions

Our case highlights an unusual presentation of spontaneous pneumomediastinum in an elderly patient without identifiable precipitating factors, associated with rapidly progressive, extensive subcutaneous emphysema requiring intervention. Although spontaneous pneumomediastinum is a rare condition that commonly affects young males and is usually benign and self-limiting, it can occasionally present atypically with extensive subcutaneous emphysema, requiring close monitoring and timely management.

Hence, spontaneous pneumomediastinum is an important differential diagnosis that should be considered in patients presenting with acute chest symptoms and subcutaneous emphysema. Prompt diagnosis and appropriate management are essential to prevent complications and ensure favorable clinical outcomes.
